# Inhibitory effect of the *Pseudobrickellia brasiliensis* (Spreng) R.M. King & H. Rob. aqueous extract on human lymphocyte proliferation and IFN-γ and TNF-α production *in vitro*

**DOI:** 10.1590/1414-431X20175163

**Published:** 2017-07-10

**Authors:** V.G. Almeida, B.A. Avelar-Freitas, M.G. Santos, L.A. Costa, T.J. Silva, W.F. Pereira, M.L.L. Amorim, C.F.F. Grael, L.E. Gregório, E. Rocha-Vieira, G.E.A. Brito-Melo

**Affiliations:** 1 Programa Multicêntrico de Pós-Graduação em Ciências Fisiológicas, Universidade Federal dos Vales do Jequitinhonha e Mucuri, Diamantina, MG, Brasil; 2 Laboratório de Imunologia, Centro Integrado de Pós-Graduação e Pesquisa em Saúde do Vale do Jequitinhonha, Universidade Federal dos Vales do Jequitinhonha e Mucuri, Diamantina, MG, Brasil; 3 Laboratório de Farmacognosia, Departamento de Farmácia, Universidade Federal dos Vales do Jequitinhonha e Mucuri, Diamantina, MG, Brasil; 4 Laboratório de Insumos Naturais e Sintéticos, Departamento de Ciências Farmacêuticas, Universidade Federal de São Paulo, Diadema, SP, Brasil

**Keywords:** Cytokines, Lymphocyte proliferation, Luteolin, Medicinal plant, Pseudobrickellia brasiliensis, Quinic acid

## Abstract

*Pseudobrickellia brasiliensis* (Asteraceae) is a plant commonly known as arnica-do-campo and belongs to the native flora of the Brazilian Cerrado. The alcoholic extract of the plant has been used as an anti-inflammatory agent in folk medicine, but the biological mechanism of action has not been elucidated. The present study evaluated the composition of *P. brasiliensis* aqueous extract and its effects on pro-inflammatory cytokine production and lymphocyte proliferation. The extracts were prepared by sequential maceration of *P. brasiliensis* leaves in ethanol, ethyl acetate, and water. Extract cytotoxicity was evaluated by trypan blue exclusion assay, and apoptosis and necrosis were measured by staining with annexin V-FITC and propidium iodide. The ethanolic (ETA) and acetate (ACE) extracts showed cytotoxic effects. The aqueous extract (AQU) was not cytotoxic. Peripheral blood mononuclear cells stimulated with phorbol myristate acetate and ionomycin and treated with AQU (100 μg/mL) showed reduced interferon (IFN)-γ and tumor necrosis factor (TNF)-α expression. AQU also inhibited lymphocyte proliferative response after nonspecific stimulation with phytohemagglutinin. The aqueous extract was analyzed by liquid chromatography coupled with photodiode array detection and mass spectrometry. Quinic acid and its derivatives 5-caffeoylquinic acid and 3,5-dicaffeoylquinic acid, as well as the flavonoids luteolin and luteolin dihexoside, were detected. All these compounds are known to exhibit anti-inflammatory activity. Taken together, these findings demonstrate that *P. brasiliensis* aqueous extract can inhibit the pro-inflammatory cytokine production and proliferative response of lymphocytes. These effects may be related to the presence of chemical substances with anti-inflammatory actions previously reported in scientific literature.

## Introduction

The use of medicinal plants as an alternative therapy is still a common practice in some regions of Brazil. However, many of these plants, though well-known in popular culture, have not been submitted to investigations for identifying their chemical compounds or assayed to determine their biological activity, which may justify their use.

*Pseudobrickellia brasiliensis* (Spreng) R.M. King & H. Rob. (Asteraceae) is a native species of the Brazilian Cerrado (1) known for its medicinal properties. The alcoholic extract obtained from the leaves of this species is used as a topical anti-inflammatory, healing, and analgesic agent and is currently an effective treatment of chronic inflammatory processes (2–5).

The inflammatory response is a complex network of biological events involving vascular changes, high expression/activity of enzymes involved in the production of lipid mediators, activation of leukocytes, and production of cytokines. These events have several functions that aim to eliminate harmful agents and restore the homeostasis of the tissue (6,7). T lymphocytes have a pivotal role in cytokine production during chronic inflammation (8,9). These cells, once activated in secondary lymphoid organs, undergo clonal proliferation, and then migrate to the injured tissue in the late stage of the inflammatory process. These cells are responsible for the mononuclear histological aspect, characteristic of the chronic inflammatory process, and contribute to the high expression of cytokines and chemokines in the inflammatory focus (10). Among the pro-inflammatory cytokines produced, interleukin (IL)-2 acts as an autocrine growth factor and induces clonal proliferation of T and B lymphocytes, and tumor necrosis factor (TNF)-α and interferon (IFN)-γ contribute to the activation of phagocytes, promote high expression levels of adhesion molecules in endothelial cells, and accelerate the events that culminate in tissue injury. In contrast, the production of anti-inflammatory cytokines such as IL-4, IL-5, and IL-10 promote regulatory or immunomodulatory functions and contribute to the control of the aggressive events observed in the long-term inflammatory process (11,12). The balance between pro- and anti-inflammatory cytokines influences the course of inflammation.

*P. brasiliensis* is used as an anti-inflammatory agent in traditional medicine. This study aimed to verify whether non-toxic *P. brasiliensis* extracts inhibit pro-inflammatory cytokine production and proliferation of human peripheral blood lymphocytes *in vitro*. The main chemical compounds of these extracts were also identified.

## Material and Methods

### Plant material, identification, and preparation of *P. brasiliensis* extract

*P. brasiliensis* leaves were collected in Diamantina, MG, Brazil (18°12,164′ South, 43°34,398′ East). The botanical identification was performed by Dr. P.A. Oliveira from the Felfili Dendrologic Herbarium (HDJF) of the Federal University of Jequitinhonha and Mucuri Valleys (UFVJM), where the specimen was deposited under voucher No. 833. *P. brasiliensis* leaves were dried and macerated sequentially in n-hexane, ethyl acetate, ethanol, and water for 72 h at room temperature, then filtered. Ethanol (ETA) and ethyl acetate (ACE) extracts were concentrated under vacuum on a rotary evaporator (Fisatom, model 801, Brazil) at 40-42°C, under reduced pressure. Aqueous extract (AQU) was filtered, frozen, and lyophilized. ACE and ETA extracts were diluted to 2.0 mg/mL with dimethyl sulfoxide (DMSO; Sigma-Aldrich Corporation, USA). AQU extract was diluted with phosphate buffered saline (PBS; 1.50 M NaCl; 0.08 M Na_2_HPO_4_; 0.02 M NaH_2_PO_4_, pH 7.4). Small stock aliquots were kept at –20°C until their use. New dilutions of the extract in DMSO or PBS were performed to obtain the required concentration in cell culture medium.

Authorization for plant collection was provided by the Brazilian Ministry of the Environment (registration No. 35605-1) and the National Council of Technological and Scientific Development (CNPq/CGEN 010832/2014-9).

### Determination of the chromatographic profile of *P. brasiliensis* extract by high-performance liquid chromatography (HPLC) and mass spectrometry (ESI-MS)

#### Sample solution

A sample solution was prepared using 10 mg of previously dried *P. brasiliensis* aqueous extract and dissolved in HPLC-grade methanol:water (1:9; 1 mL) on the sonication bath for 5 min. The solution was filtered through a 0.2 µm pore size syringe filter with a diameter of 13 mm.

#### Chromatographic system

The ultra-fast liquid chromatography (UFLC) system used consisted of a Shimadzu Prominence UFLC system (Japan) equipped with two pumps (LC-20AD), an on-line degasser (DGU-20A), a column oven (CTO-20A), an autosampler (SIL-20AHT), and a PDA detector (SPD-20A) coupled with a TOF mass spectrometry detector (micrOTOF-Q™; Bruker Daltonics, USA). It used a VP-ODS (Shimadzu) analytical column (150×2 mm, particle size 4.6 µm) with a GPV-ODS guard column (5×2 mm; Shimadzu). Ultrapure water (Type 1; Direct-Q® 5 System, Merck Millipore, USA) and acetonitrile (HPLC grade) were used as the mobile phase. Analysis was performed at a flow rate of 0.8 mL/min and monitored at 190–600 nm.

The gradient mobile phases used were as follows: 0–5 min: water (90%), acetonitrile (10%); 45–50 min: acetonitrile (100%), and 52–60 min: water (90%) and acetonitrile (10%).

#### Direct infusion ESI-MS and ESI-MS/MS analyses

Direct infusion ESI-MS and ESI-MS/MS analyses were performed using a Bruker Daltonics micrOTOF-Q™ II, ESI-Qq-TOF mass spectrometer system operating in negative ion mode.

### Biological samples and peripheral blood mononuclear cell (PBMC) separation

PBMCs were obtained from 31 healthy adult blood donors (10 men and 21 women; 25±5 years old). Subjects with any infectious or autoimmune disease, or those using antibiotics, anti-inflammatory drugs, corticosteroids, or other immunosuppressive drugs were not considered for blood donation. Informed written consent was obtained from all participants. The study was approved by the Ethical Committee of the UFVJM, Diamantina, MG, Brazil (register code 02/009). Blood samples were collected in vacuum tubes containing heparin (Vacutainer; Becton Dickinson, USA).

PBMCs were isolated from heparinized human peripheral blood samples by the Ficoll-Histopaque (specific gravity, 1.077) gradient density method (13). The peripheral blood (15 mL) was diluted with PBS and centrifuged in a Ficoll-Histopaque 1.077 (Sigma-Aldrich Corporation, USA) gradient at 400 *g* at room temperature for 30 min. PBMCs were collected, washed with PBS, and centrifuged (240 *g*, 7 min). Cells were re-suspended at a concentration of 1×10^7^ cells/mL in PBS or RPMI-1640 medium (Sigma-Aldrich Corporation) supplemented with 10% heat-inactivated fetal calf serum (FCS; Gibco, Invitrogen Corporation, USA), 2 mM L-glutamine (Sigma-Aldrich Corporation), and antibiotic/antimycotic cocktail (penicillin G 100 IU/mL, streptomycin 100 µg/mL and amphotericin B 250 ng/mL; Sigma-Aldrich Corporation).

### Cell viability analyses

PBMCs (5×10^5^; n=4) were cultured in RPMI-1640 medium (Sigma-Aldrich Corporation) supplemented with 2 mM L-glutamine, 10% FCS (Gibco, Invitrogen Corporation), and antibiotic-antimycotic cocktail (penicillin G 100 IU/mL, streptomycin 100 μg/mL and amphotericin B 250 ng/mL, Sigma-Aldrich Corporation). Cells were treated with ACE, ETA, or AQU (12.5, 25, 50, or 100 μg/mL) extracts or an equal volume of DMSO (Sigma-Aldrich Corporation) (solvent control) in a humidified incubator at 37°C with 5% CO_2_ air atmosphere for 24 h or 5 days. Untreated PBMCs constituted the cell culture control (CON). PBMCs treated with cadmium chloride (CdCl_2_; 20mM) constituted the positive control of cell death. PBMCs were washed with PBS (200 *g*, 7 min, 4°C) and re-suspended in 0.5 mL PBS. Then, 10 μL cell suspension was mixed with 190 μL of 0.002% trypan blue (Sigma-Aldrich Corporation) and analyzed by flow cytometry (FACScan, Becton Dickinson, USA) (14). Ten thousand events were acquired in the region corresponding to lymphocytes. CellQuest™ software (Becton Dickinson) was used for data collection and analyses. Cell viability was calculated by dividing the number of viable cells by the total cell number.

The evaluation of apoptosis or necrosis induced by *P. brasiliensis* extract was performed by flow cytometry using a commercial kit for apoptotic cell detection (Annexin V FITC Apoptosis Detection Kit II, BD Pharmingen, USA). PBMCs (5×10^5^; n=8) were incubated in RPMI-1640 containing ACE, ETA, or AQU (50 μg/mL) extracts or an equal volume of DMSO (solvent control) at 37°C in a humidified incubator with a 5% CO_2_ air atmosphere for 4 h. Untreated PBMCs constituted the negative cell culture control (CON). Cells were washed (200 *g*, 10 min, 4°C) and re-suspended in 100 µL binding buffer. Cells were incubated with 2 µL of annexin V-FITC and 2 µL of propidium iodide, then analyzed by flow cytometry (FACScan, Becton Dickinson). At least 20,000 events were acquired in the region corresponding to lymphocytes. CellQuest™ software (Becton Dickinson) was used for data collection and analyses.

### Effect of *P. brasiliensis* aqueous extract on cytokine production

PBMCs (5×10^5^) (n=8) were cultured in RPMI-1640 containing 10% FCS (Gibco, Invitrogen Corporation), 2 mM L-glutamine, antibiotic-antimycotic cocktail (Sigma-Aldrich Corporation), 25 ng/mL phorbol-12-myristate-13-acetate-PMA (Sigma-Aldrich Corporation), 1 ng/mL ionomycin (Sigma-Aldrich Corporation), 1 μg/mL Brefeldin-A (Sigma-Aldrich Corporation), and either PBS (negative control) or AQU (25, 50, or 100 μg/mL). Untreated PBMCs were used as the non-stimulated cell culture control (CON). The cells were maintained at 37°C in a humidified incubator with a 5% CO_2_ air atmosphere for 4 h. Cells were then fixed, permeabilized, and incubated with the monoclonal antibody (mAb) phycoerythrin (PE) conjugated specifically for IFN-γ (phycoerythrin-conjugated IFN-γ mAb-IFN-γ-PE, clone 45.B3), TNF-α (phycoerythrin-conjugated TNF-α mAb-TNF-α-PE, clone MAb11), and IL-10 (phycoerythrin-conjugated IL-10 mAb-IL10-α-PE, clone JES3-9D7) cytokines (all from Biolegend, USA). The expression of cytokines by lymphocytes was evaluated by flow cytometry (FACScan, Becton Dickinson). At least 30,000 events were acquired in a region corresponding to the PBMCs. The collection and analyses of data were performed with CellQuest™ software (Becton Dickinson).

### Effect of *P. brasiliensis* aqueous extract on the lymphocyte proliferative response

The effect of AQU on the proliferative lymphocyte response to PHA stimulation was evaluated by 5-(and-6)-carboxyfluorescein succinimidyl ester (CFSE) fluorescence decay assay (15). PBMCs (1×10^7^) were re-suspended in PBS/BSA 0.1% and labeled with 10 μM CFSE (Sigma-Aldrich Corporation) for 10 min at 37°C. CFSE-stained PBMCs (5×10^5^; n=6) were cultured in RPMI-1640 containing 10% FCS (Gibco, Invitrogen Corporation), 2 mM L-glutamine, antibiotic-antimycotic cocktail (Sigma-Aldrich Corporation), either with or without phytohemagglutinin (PHA) (1 μg/mL). Cells also were stimulated with PHA in combination with different concentrations of AQU (25, 50, or 100 μg/mL). Untreated PBMCs were used as the non-stimulated cell culture control (CON). The cells were kept in a humidified incubator with a 5% CO_2_ air atmosphere for 5 days at 37°C. After incubation, the cells were harvested, washed in PBS, and stained with specific mAb for human CD3 (anti-CD3 biotin, clone HIT3a), CD4 (CD4 mAb phycoerythrin-cyanine conjugated - CD4/PeCy5, clone RPA-T4), and CD8 (CD8 mAb phycoerythrin-cyanine conjugated - CD8/PeCy5, clone RPA-T8) (both from BD Biosciences, USA). After washing with PBS (0.015 M PBS, pH 7.4, 0.5% BSA and 0.1% sodium azide), the suspensions were incubated for 15 min in the dark at 4°C with streptavidin-phycoerythrin conjugated (BD Biosciences). Samples were analyzed on a BD FACScan with CellQuest™ software, and a total of 50,000 events were acquired for each tube. Proliferation was measured on the basis of the dilution of CFSE (diminished staining intensity). The proliferative index was then calculated from CFSE fluorescence histograms using the following formula (16): Proliferative index = (100-Y)/Y, where Y (%) = X0+X1/2+X2/4+X3/8+X4/16+X5/32+X6/64; X0 represents the percentage of T cells that did not divide and X1-6 represent the maximum gradual division.

### Statistical analyses

GraphPad Prism, version 5.0 for Windows (GraphPad Software, USA) was used for statistical analysis. One-way ANOVA and Tukey's *post hoc* test were used for analyses. P values lower than 0.05 were considered to be statistically significant. Data are reported as means±SD.

## Results and Discussion

**Figure 1.**
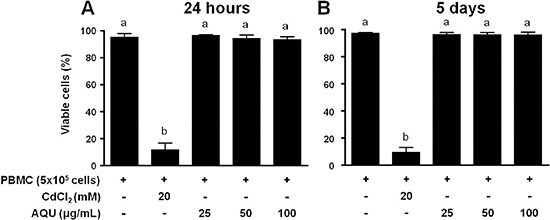
Analysis of the cytotoxic effect of *P. brasiliensis* aqueous extract with the trypan blue exclusion assay. Percentage of viable cells in cultures of peripheral blood mononuclear cells (PBMCs) treated with or without *P. brasiliensis* aqueous extract (AQU) after 24 h (*A*) or 5 days (*B*) of incubation. Evaluation was done with trypan blue exclusion assay (n=8). Culture treated with cadmium chloride (CdCl_2_) constituted the positive control of cell death. Results are reported as means±SD. Different letters indicate significant differences (P≤0.05, ANOVA with Tukey *post hoc*).

**Figure 2.**
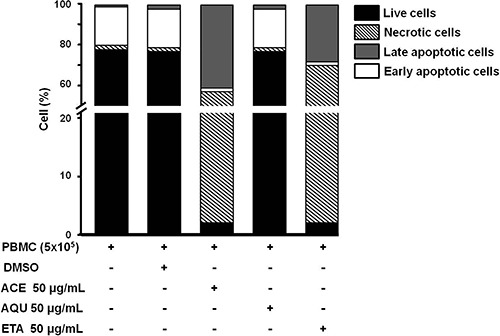
Apoptotic or necrotic effect of *P. brasiliensis* extracts. Percentage of viable, apoptotic, or necrotic cells in cultures of peripheral blood mononuclear cells (PBMCs) treated with or without *P. brasiliensis* extracts after 4-h incubation. Evaluation was done by flow cytometry (n=8). Culture treated with dimethyl sulfoxide (DMSO) constituted the solvent control. ACE: *P. brasiliensis* ethyl acetate extract; AQU: *P. brasiliensis* aqueous extract; ETA: *P. brasiliensis* ethanolic extract.

Since plant extracts and solvents are considered external factors that can directly influence cell death or activate mechanisms linked to programmed cell death (17,18), we initially investigated the cytotoxicity of the *P. brasiliensis* extracts through cell viability and apoptosis/necrosis analyses in cell cultures treated with different extracts of the plant. At different concentrations, the AQU extract did not affect cell viability after 24 h (Figure 1A) or 5 days (Figure 1B), compared to the viability of CON cultures. ETA and ACE reduced cell viability to about 0.3% after 24 h, relative to control cell cultures (data not shown). Although the trypan blue exclusion test is a technique widely used to evaluate cell viability, this assay does not detect cell modifications observed in the early stages of cell death. Therefore, in an attempt to determine whether the presence of the plant extracts could trigger early apoptotic/necrotic mechanisms, we performed an additional cell analysis using propidium iodide and annexin V. The cell viability of the cultures treated with 50 μg/mL AQU did not significantly differ from that of CON culture (Figure 2). In contrast, cell cultures treated with 50 µg/mL ACE and ETA showed a low percentage of viable cells and a high percentage of necrotic and apoptotic lymphocytes, in comparison to the CON and DMSO cultures. These data are in agreement with the cell viability data obtained with the trypan blue assay, and allow us to conclude that toxicity demonstrated by ACE and ETA is not attributable to the solvent used for dilution. Moreover, the treatment with AQU extract showed no toxicity to lymphocytes *in vitro*. Assays to evaluate the cytotoxic activity of medicinal plants *in vitro* are essential to assess the continuity of subsequent experimental steps. Therefore, based on cell toxicity, the sequential analyses of the chemical compounds and immune parameters were performed using only the AQU extract.

**Figure 3.**
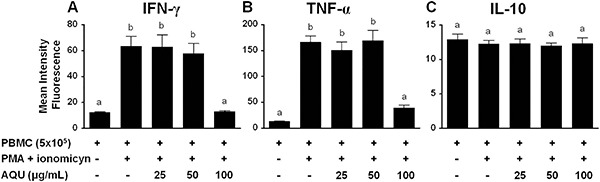
Effect of *P. brasiliensis* aqueous extract on cytokine production by lymphocytes. Peripheral blood mononuclear cells (PBMCs, 5×10^5^ cells; n=8) were stimulated with phorbol myristate acetate (PMA) in the presence of 25, 50, or 100 µg/mL of *P. brasiliensis* aqueous extract (AQU). Non-stimulated cultures constituted the negative control (PBMC), and cultures stimulated with PMA + ionomycin in the absence of plant extract constituted the positive control. Production of IFN-γ (*A*), TNF-α (*B*), and IL-10 (*C*) was determined by flow cytometry. Results are reported as means±SD. Different letters indicate significant differences (P≤0.05, ANOVA with Tukey *post hoc*).

Regarding the balance between pro- and anti-inflammatory cytokines and its influence on the course of inflammation mediated by T lymphocytes, we decided to evaluate TNF-α, IFN-γ, and IL-10 production by PMA-stimulated lymphocytes using a flow cytometry platform. The results were presented as mean fluorescence intensity (MFI), which allows the correlation of the signal emitted by fluorescent-conjugated specific monoclonal antibodies and the cytokine expression by specific cells. Three different concentrations of AQU (25, 50, and 100 μg/mL) were used to verify the inhibitory effect of the plant extract on the pro-inflammatory cytokine production. According to the results, the expression of IFN-γ in lymphocytes was 4.8-fold lower in cultures treated with PMA plus AQU (100 μg/mL) than in cultures treated with PMA alone (Figure 3A). Similarly, in the cell cultures treated with 100 µg/mL AQU, the TNF-α expression was 3.8-fold lower than in the PMA-stimulated cell cultures (Figure 3B). There were no differences in the IL-10 production by lymphocytes between cell cultures submitted to different experimental conditions. In summary, the *P. brasiliensis* aqueous extract exerted a strong inhibitory action on pro-inflammatory cytokine production in stimulated T lymphocytes *in vitro*.

**Figure 4.**
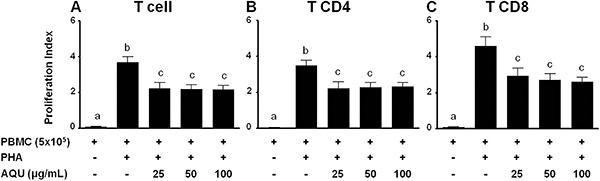
Antiproliferative effect of *P. brasiliensis* aqueous extract. Peripheral blood mononuclear cells (PBMCs, 5×0^5^ cells; n=6) labeled with carboxyfluorescein succinimidyl ester (CFSE) were stimulated with phytohemagglutinin (PHA) in the absence or presence (25, 50, or 100 µg/mL) of *P. brasiliensis* aqueous extract (AQU). Non-stimulated cultures constituted the negative control (PBMC), and cultures stimulated with PHA in the absence of plant extract constituted the positive control. After 5 days, the proliferation index of lymphocytes (*A)*, CD4+ T cells (*B*), and CD8+ T cells (*C*) was calculated by cytometry analysis. Data are reported as means±SD. Different letters indicate significant differences (P≤0.05, ANOVA with Tukey *post hoc*).

Because lymphocyte proliferation is a pivotal and essential event in immune-mediated inflammatory response (19), we also evaluated the proliferative response of cells treated with AQU extract. All tested concentrations of the AQU extract reduced the proliferative response of T lymphocytes, including CD4+ and CD8+ cells (Figure 4). These data support the finding that AQU extract exhibits an anti-inflammatory effect *in vitro*, and strengthens the validity of the data collected in the cytokine analyses. The inhibitory effects observed suggest that AQU extract components may interfere with cellular activation mechanisms directly involved in cytokine production and lymphocyte capacity expansion.

The results obtained in our biological assays of the AQU extract of the plant revealed an anti-proliferative action on human lymphocytes and an inhibitory effect on pro-inflammatory cytokine production *in vitro*. Chemical analysis of AQU extract was carried out to verify the presence of chemical compounds with anti-inflammatory activities that have already been described in the literature. Previous studies with *P. brasiliensis* apolar extracts revealed the presence of triterpenes and sesquiterpenes, such as hidroxigermacreno, lupeol, β-amyrin acetate, spathulenol, cadinene, cadinol, oplopanona acetate, and α-acetoxy-β-amyrin (20). Moreover, the essential oil from the leaves contains the major compounds terpinen-4-ol, γ-terpinene, α-terpinene, and α-terpineol (21). However, no study has been conducted to identify the chemical compounds present in the polar extract of *P. brasiliensis*, a similar formulation used in folk medicine.

**Figure 5.**
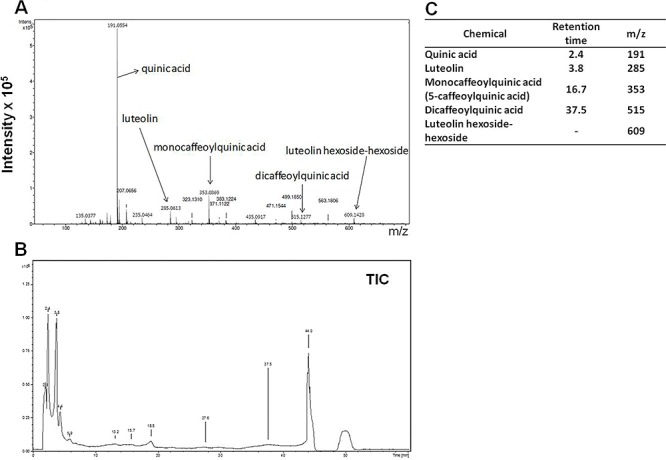
Representative ESI-MS and LC-PDA-MS analysis of the aqueous extract of *Pseudobrickellia brasiliensis*. *A*, ESI-MS full scan; *B*, total ion chromatogram (TIC), and *C*, chemical, retention time (in min), and *m/z.*

To identify the chemical components in the AQU extract, we performed LC-PDA-MS, ESI-MS, and ESI-MS/MS (direct infusion) analyses (Figure 5). The results indicated the presence of quinic acid and its derivatives, 5-caffeoylquinic acid and 3,5-dicaffeoylquinic acid. These derivatives are known as chlorogenic acids, which are a variety of polyphenolic acids present in food and medicinal plants. Furthermore, the presence of the ions *m/z* 609 and *m/z* 285 suggests the presence of the flavonoid luteolin (*m/z* 285) and its derivative luteolin dihexoside (*m/z* 609).

Hepatoprotective, antioxidant, anti-inflammatory, and analgesic actions of quinic acid and its derivatives have been previously described (22–26). The quinic acid derivative obtained from *Pimpinella brachycarpa* (Umbeliferae) showed an inhibitory effect against nitric oxide (NO) production in microglia (26). Chlorogenic acids, including 5-caffeoylquinic and dihidrocafeilquinic acids, are among the main constituents of the aqueous extract of Brazilian green propolis collected from *Apis mellifera* hives. This extract showed an anti-inflammatory effect in rats that received both subcutaneous implantation of polyester-polyurethane sponges cut into disks and oral treatment with 500 mg/kg of green propolis (27).

The *in vitro* anti-inflammatory effects of 5-o-caffeoylquinic acid were demonstrated by its inhibitory action on NO production, expression of COX-2 and iNOS, and production of pro-inflammatory cytokines (TNF-α, IL-1β, and IL-6) and adhesion molecules in LPS-stimulated murine microglia/macrophages. It was also demonstrated that 5-o-caffeoylquinic acid inhibited the nuclear translocation of NF-κB (28).

Quinic acid derivatives, including 3,5-dicaffeoylquinic acid, are also present in *Lychnophora ericoides* (Asteraceae), popularly known as arnica-da-serra. The 3,5-dicaffeoylquinic acid purified from the polar extract of *L. ericoides* demonstrated analgesic activity in animal models of acetic acid-induced abdominal contortion (22).

Luteolin is found in various plants of the Asteraceae family that exhibit anti-inflammatory activity. It is a flavonoid with potent anti-inflammatory activity both *in vitro* and *in vivo* (29–31), and anti-carcinogenic (32) and antioxidant (33,34) proprieties. The anti-inflammatory action of luteolin was demonstrated through its inhibitory effect on the expression of pro-inflammatory cytokine TNF-α via the inhibition of the NF-kB signaling pathway (35). Furthermore, luteolin inhibits the expression of IL-6 by inhibiting the JNK and AP-1 pathways (36,37). The anti-inflammatory effect of luteolin may also be related to its inhibitory effect on COX-2, LOX, and iNOS enzymes (29,38,39).

Although the inhibitory effect of AQU on T lymphocyte proliferation and pro-inflammatory cytokine production was not associated specifically with the compounds identified in our study, the presence of active molecules linked to anti-inflammatory action already described in literature provides strong evidence for the therapeutic potential of the plant. Many phytomedicines are composed of a mixture of several components, exerting their therapeutic effects through the synergistic action of compounds. Further studies with fractionated AQU extract will be performed to elucidate the structure of the substances responsible for the effect demonstrated here, as well as the performance of the active fractions in inflammatory models *in vivo*.

In summary, our results showed that the aqueous extract of *P. brasiliensis* demonstrated no cytotoxic activity at the tested concentrations. It exerted an anti-inflammatory effect *in vitro* by decreasing the expression of the pro-inflammatory cytokines TNF-α and IFN-γ and by inhibiting lymphocyte proliferation. These data suggest that some of the therapeutic effects of *P. brasiliensis* may be because of its actions on essential mechanisms for inflammation maintenance.

## References

[1] Nakajima JN, Semir J (2016). Asteraceae do Parque Nacional da Serra da Canastra, Minas Gerais, Brasil. Braz J Bot.

[2] Ribeiro JF, Felfili JM, Walter BMT, Mendonça RC, Filgueiras TS, Silva MR (2001). Caracterização florística e potencial de uso das espécies vasculares ocorrentes nas fazendas Trijunção, BA.

[3] Souza FAA (2006). A cultura tradicional do sertanejo e o seu deslocamento para a implantação do Parque Nacional Grande Sertão Veredas.

[4] Reis SLA, Bellini LM Conhecimento e uso da flora para fins medicinais em comunidades ribeirinhas dos rios Paraná, PR e Cuiabá, MT.

[5] Carneiro MRB (2009). A Flora medicinal no Centro Oeste do Brasil: um estudo de caso com abordagem etnobotânica em Campo Limpo de Goiás.

[6] Gilroy DW, Lawrence T, Perretti M, Rossi AG (2004). Inflammatory resolution: new opportunities for drug discovery. Nat Rev Drug Discov.

[7] Medzhitov R (2008). Origin and physiological roles of inflammation. Nature.

[8] Ganguly T, Badheka LP, Sainis KB (2001). Immunomodulatory effect of Tylophora indica on Con A induced lymphoproliferation. Phytomedicine.

[9] Goncharova LB, Tarakanov AO (2007). Molecular networks of brain and immunity. Brain Res Rev.

[10] Guy CS, Vignali KM, Temirov J, Bettini ML, Overacre AE, Smeltzer M (2013). Distinct TCR signaling pathways drive proliferation and cytokine production in T cells. Nat Immunol.

[11] Zhang JM, An J (2007). Cytokines, inflammation, and pain. Int Anesthesiol Clin.

[12] de Oliveira CM, Sakata RK, Issy AM, Gerola LR, Salomao R (2011). Cytokines and pain. Rev Bras Anestesiol.

[13] Bicalho HMS, Gontijo MC, Nogueira-Machado JA (1981). A simple technique for simultaneous human leukocytes separation. J Immunol Meth.

[14] Avelar-Freitas BA, Almeida VG, Pinto MC, Mourao FA, Massensini AR, Martins-Filho OA (2014). Trypan blue exclusion assay by flow cytometry. Braz J Med Biol Res.

[15] Lyons AB (2000). Analysing cell division *in vivo* and *in vitro* using flow cytometric measurement of CFSE dye dilution. J Immunol Methods.

[16] Angulo R, Fulcher DA (1998). Measurement of Candida-specific blastogenesis: comparison of carboxyfluorescein succinimidyl ester labelling of T cells, thymidine incorporation, and CD69 expression. Cytometry.

[17] Parolin MB, Reason IJ (2001). [Apoptosis as a mechanism of tissue injury in hepatobiliary diseases]. Arq Gastroenterol.

[18] Grivicich I, Regner A, Rocha AB (2007). Apoptosis: programmed cell death. Rev Bras Cancerol.

[19] Boyman JF, Purton CD, Surh J, Sprent (2007). Cytokines and T-cell homeostasis. Curr Opin Immunol.

[20] Bohlmann F, Zdero C, King RM, Robinson H (1984). A hydroxygermacrene and other constituents from *Pseudobrickellia brasiliensis*. Phytochemistry.

[21] Silva RF, Rezende CM, Pereira JB, Vieira RF, Santos MCS, Bizzo HR (2015). Scents from Brazilian Cerrado: chemical composition of the essential oil from *Pseudobrickellia brasiliensis* (Asteraceae). J Essent Oil Res.

[22] Dos Santos MD, Gobbo-Neto L, Albarella L, de Souza GE, Lopes NP (2005). Analgesic activity of di-caffeoylquinic acids from roots of *Lychnophora ericoides* (Arnica da serra). J Ethnopharmacol.

[23] Kim KH, Kim YH, Lee KR (2007). Isolation of quinic acid derivatives and flavonoids from the aerial parts of *Lactuca indica* L. and their hepatoprotective activity *in vitro*. Bioorg Med Chem Lett.

[24] An RB, Sohn DH, Jeong GS, Kim YC (2008). In vitro hepatoprotective compounds from Suaeda glauca. Arch Pharm Res.

[25] Dos Santos MD, Chen G, Almeida MC, Soares DM, de Souza GE, Lopes NP (2010). Effects of caffeoylquinic acid derivatives and C-flavonoid from *Lychnophora ericoides* on *in vitro* inflammatory mediator production. Nat Prod Commun.

[26] Lee SY, Moon E, Kimb SY, Lee KR (2013). Quinic acid derivatives from *Pimpinella brachycarpa* exert anti-neuroin?ammatory activity in lipopolysaccharide-induced microglia. Bioorg Med Chem Lett.

[27] Moura SAL, Negri G, Salatino A, Lima LDC, Dourado LPA, Mendes JB (2011). Aqueous extract of Brazilian green propolis: primary components, evaluation of inflammation and wound healing by using subcutaneous implanted sponges. J Evid Based Complementary Altern Med.

[28] Hwang SJ, Kim YW, Park Y, Lee HJ, Kim KW (2014). Anti-inflammatory effects of chlorogenic acid in lipopolysaccharide-stimulated RAW 264.7 cells. Inflamm Res.

[29] Chen CY, Peng WH, Tsai KD, Hsu SL (2007). Luteolin suppresses inflammation-associated gene expression by blocking NF-kappaB and AP-1 activation pathway in mouse alveolar macrophages. Life Sci.

[30] Kang OH, Choi JG, Lee JH, Kwon DY (2010). Luteolin isolated from the flowers of Lonicera japonica suppresses inflammatory mediator release by blocking NF-kappaB and MAPKs activation pathways in HMC-1 cells. Molecules.

[31] Jeon IH, Kim HS, Kang HJ, Lee HS, Jeong SI, Kim SJ (2014). Anti-inflammatory and antipruritic effects of luteolin from Perilla (*P.* frutescens L.) leaves. Molecules.

[32] Wang L, Li W, Lin M, Garcia M, Mulholland D, Lilly M (2014). Luteolin, ellagic acid and punicic acid are natural products that inhibit prostate cancer metastasis. Carcinogenesis.

[33] Manju V, Balasubramaniyan V, Nalini N (2005). Rat colonic lipid peroxidation and antioxidant status: the effects of dietary luteolin on 1,2-dimethylhydrazine challenge. Cell Mol Biol Lett.

[34] Seelinger G, Merfort I, Schempp CM (2008). Anti-oxidant, anti-inflammatory and anti-allergic activities of luteolin. Planta Med.

[35] Lv L, Lv L, Zhang Y, Kong Q (2011). Luteolin prevents LPS-induced TNF-alpha expression in cardiac myocytes through inhibiting NF-kappaB signaling pathway. Inflammation.

[36] Li YC, Yeh CH, Yang ML, Kuan YH (2012). Luteolin suppresses inflammatory mediator expression by blocking the Akt/NFκB pathwayin acute lung injury induced by lipopolysaccharide in mice. Evid Based Complement Alternat Med.

[37] Park CM, Song YS (2013). Luteolin and luteolin-7-O-glucoside inhibit lipopolysaccharide-induced inflammatory responses through modulation of NF-kappaB/AP-1/PI3K-Akt signaling cascades in RAW 264.7 cells. Nutr Res Pract.

[38] Hu C, Kitts DD (2004). Luteolin and luteolin-7-O-glucoside from dandelion flower suppress iNOS and COX-2 in RAW264.7 cells. Mol Cell Biochem.

[39] Ziyan L, Yongmei Z, Nan Z, Ning T, Baolin L (2007). Evaluation of the anti-inflammatory activity of luteolin in experimental animal models. Planta Med.

